# Supplementary material for: Mucin-type O-glycans regulate proteoglycan stability and chondrocyte maturation
Source: bioRxiv. 2025 Dec 14:2025.12.11.693745. Preprint. [Version 2] doi: 10.64898/2025.12.11.693745 (PMC12710741; doi:10.64898/2025.12.11.693745)
Supplement: Supplement 1 [file media-1.pdf]

Supplementary Information for:

Mucin-type *O*-glycans regulate proteoglycan stability and chondrocyte maturation

Xiaolin Dong[1,2], Sydney Bedillion[1,2], Kaleigh E. Gosnell [2], Peng Zhao[1], Amrita Basu[1], Kavya Suryadevara[2], Digantkumar Chapla[1], Lance Wells[1,2], Ryan J. Weiss[1,2]*

*Correspondence should be addressed to R.J.W.
Email: ryan.weiss@uga.edu

This PDF file includes:

Supplementary Tables 1-9
Supplementary Figures 8-9

Supplementary Table 1:
sgRNA sequences used for CRISPR/Cas9 gene editing

Gene	Species	Guide Sequence (5’→3’)
Non-targeting	–	ACGTTCGAGTACGACCAGCT
COSMC (C1GALT1C1)	Human	ATGCTAGGACACATTAGGAT
C1GALT1	Human	TTTAAGCCTTATGTAAAGCA
Cosmc (C1galt1c1)	Mouse	ATATGGACACAAATGACATG
C1galt1	Mouse	GTGAAGCAGGGATACATGAG
CD44	Human	CATCACGGTTAACAATAGCT

Supplementary Table 2:
PCR primer sequences used for genotyping CRISPR/Cas9 engineered cells

Gene	Forward Primer (5’→3’)	Reverse Primer (3’→5’)
COSMC	TGATTTCAAGCTTGGGAACCTTT	CATCCCTCCCTGTTCAGGAC
C1GALT1	CCCTGCTGTGGGACTGAAAA	GGTGTTCTGGCACAAAGGGA
CD44	ACATAGACGTTGCTGAGAACC	TTCCATGCACCTTCCCAGAG
Cosmc (m)	AGGGAAACAAGTGGGAGAATGA	TCCCTGAATGGCATCACCAG
C1galt1 (m)	ACCATCTGCAAGCCCCTAA	TACTCCGGCGTATTTCAGGC

Supplementary Table 3:
Primer sequences used for quantitative PCR analysis of gene expression

Gene	Forward Primer Sequence (5’→3’)	Reverse Primer Sequence (3’→5’)
YWHAZ	CCTGCATGAAGTCTGTAACTGAG	GACCTACGGGCTCCTACAACA
SDC2	GCTGTTGGTGTATCGCATGA	ACTGGATGGTTTGCGTTCTC
SULF1	GAAGGAGAAGAGACGGCAGA	CAGAAAGATCCCAGGTTCCA
SULF2	CACTGGCAAGTACGTCCACAA	CTATTGAGGTACACCCCAAAGG
ESM1	GAGAAACTTGCTACCGCACA	CCCATTAGAAGGCTGACACC
Gapdh (m)	GGTGCTGAGTATGTCGTGGA	CCTTCCACAATGCCAAAGTT
Sox9 (m)	AGTACCCGCATCTGCACAAC	TACTTGTAATCGGGGTGGTCTTTC
Col2a1 (m)	AGGGCAACAGCAGGTTCACATAC	TGTCCACACCAAATTCCTGTTCA
Acan (m)	GCTGCAGTGATCTCAGAAGAAG	GATGGTGAGGGAAGACCCTA

Supplementary Table 4:
HS disaccharide composition for TC28a2 WT, *COSMC* KO, and *C1GALT1* KO cells

Disaccharide Structure		Abundance (% Total Disaccharide)[c]		
**Structure Code[a]**	**Unit Formula[b]**	**WT (% Total HS)**	***COSMC*$^{-/-}$ (% Total HS)**	***C1GALT1*$^{-/-}$ (% Total HS)**
D0A0	ΔUA-GlcNAc	64.97 ± 4.50	72.48 ± 2.34	65.50 ± 0.83
D0H6	ΔUA-GlcNH$_2$6S	-	-	-
D2H0	ΔUA2S-GlcNH$_2$	-	-	-
D0S0	ΔUA-GlcNS	21.13 ± 1.27	18.72 ± 0.81	22.13 ± 0.86
D0A6	ΔUA-GlcNAc6S	2.38 ± 0.35	0.83 ± 0.58	1.90 ± 0.09
D2A0	ΔUA2S-GlcNAc	1.35 ± 0.52	0.67 ± 0.15	0.72 ± 0.48
D2H6	ΔUA2S-GlcNH$_2$6S	0.31 ± 0.12	0.36 ± 0.20	0.39 ± 0.14
D0S6	ΔUA-GlcNS6S	3.06 ± 0.85	2.14 ± 0.38	2.61 ± 0.34
D2S0	ΔUA2S-GlcNS	5.13 ± 1.55	3.70 ± 0.67	4.99 ± 0.51
D2A6	ΔUA2S-GlcNAc6S	-	-	-
D2S6	ΔUA2S-GlcNS6S	1.40 ± 0.32	1.07 ± 0.11	1.63 ± 0.19

[a] The disaccharide structure code is described in (Lawrence, et al. Nat. Methods 2008)
[b] ΔUA = 4,5-unsaturated uronic acid
[c] –, not detected

Supplementary Table 5:
HS sulfation and *N*-substitution of glucosamine units for TC28a2 WT, *COSMC* KO, and *C1GALT1* KO cells

HS Sulfation	Constituents/100 disaccharides		
	**WT**	***COSMC*$^{-/-}$**	***C1GALT1*$^{-/-}$**
Unsubstituted glucosamine	65.16 ± 4.47	70.74 ± 1.22	65.48 ± 0.74
N-sulfoglucosamine	30.76 ± 3.94	26.83 ± 0.95	31.39 ± 1.42
Uronyl-2-*O*-sulfates	8.23 ± 2.44	6.58 ± 0.65	7.86 ± 0.31
Glucosaminyl 6-*O*-sulfates	7.24 ± 1.26	5.15 ± 0.64	6.68 ± 0.28

Supplementary Table 6:
HS sulfate groups per disaccharide of glucosamine units for TC28a2 WT, *COSMC* KO, and *C1GALT1* KO cells

HS Sulfates/disaccharide	Abundance (% Total Disaccharide)		
	**WT**	***COSMC**$^{-/-}$*	***C1GALT1**$^{-/-}$*
0 SO_3	65.20 ± 4.48	73.29 ± 5.57	65.60 ± 0.85
1 SO_3	24.87 ± 1.72	19.78 ± 2.98	24.77 ± 0.59
2 SO_3	8.53 ± 2.48	6.01 ± 2.40	8.00 ± 0.79
3 SO_3	1.40 ± 0.32	0.92 ± 0.33	1.63 ± 0.19

Supplementary Table 7:
CS/DS disaccharide composition of TC28a2 WT, *COSMC* KO, and *C1GALT1* KO cells

Disaccharide Structure		Abundance (% Total Disaccharide)		
**Structure Code**[a]	**Unit Formula**[b]	**WT (% Total CS)**	***COSMC**$^{-/-}$* **(% Total CS)**	***C1GALT1**$^{-/-}$* **(% Total CS)**
D0a0	ΔUA-GalNAc	6.32 ± 2.89	12.29 ± 2.75	16.44 ± 4.21
D2a0	ΔUA2S-GalNAc	3.76 ± 0.97	2.35 ± 0.37	1.83 ± 0.15
D0a6	ΔUA-GalNAc6S	28.44 ± 1.04	24.31 ± 4.84	20.07 ± 2.08
D0a4	ΔUA-GalNAc4S	50.68 ± 0.99	51.04 ± 0.98	51.16 ± 0.48
D2a6	ΔUA2S-GalNAc6S	3.28 ± 0.41	2.68 ± 0.21	3.24 ± 0.31
D2a4	ΔUA2S-GalNAc4S	2.98 ± 0.82	2.69 ± 0.55	3.42 ± 0.42
D0a10	ΔUA-GalNAc4S6S	4.53 ± 0.89	4.65 ± 1.48	3.83 ± 2.47

Supplementary Table 8:
CS/DS sulfation of *N*-acetyl-galactosamine units in TC28a2 WT, *COSMC* KO, and *C1GALT1* KO cells

CS/DS Sulfation	Abundance (% Total Disaccharide)		
	**WT**	***COSMC**$^{-/-}$*	***C1GALT1**$^{-/-}$*
Unsubstituted *N*-acetyl-galactosamine	6.32 ± 2.89	12.29 ± 2.75	16.44 ± 4.21
Uronyl-2-*O*-sulfates	6.27 ± 1.22	5.36 ± 0.46	6.66 ± 0.56
GalNAc 4-*O*-sulfates	58.16 ± 2.20	57.54 ± 1.56	57.08 ± 1.88
GalNAc 6-*O*-sulfates	40.02 ± 1.23	33.98 ± 4.19	28.99 ± 4.57

Supplementary Table 9:
CS/DS sulfate groups per disaccharide of *N*-acetyl-galactosamine units in Tc28a2 WT, *COSMC* KO, and *C1GALT1* KO cells

CS/DS Sulfation	Abundance (% Total Disaccharide)[a]		
	WT	*COSMC*$^{-/-}$	*C1GALT1*$^{-/-}$
0 SO_3	6.32 ± 2.89	12.29 ± 2.75	16.44 ± 4.21
1 SO_3	82.88 ± 1.56	77.70 ± 4.38	73.07 ± 1.69
2 SO_3	10.81 ± 2.11	10.01 ± 1.91	10.49 ± 2.77
3 SO_3	-	-	-

[a] –, not detected

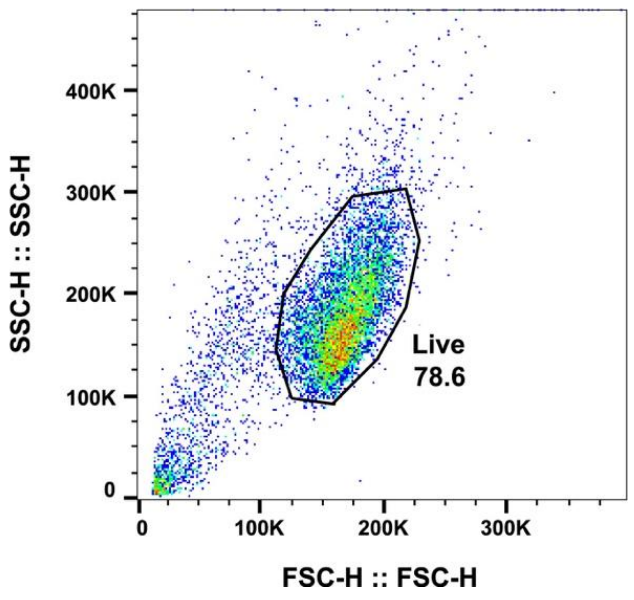

Supplementary Figure 8. General flow cytometry gating strategy. Cells were gated based on forward and side scattering for analysis of flow cytometry data.

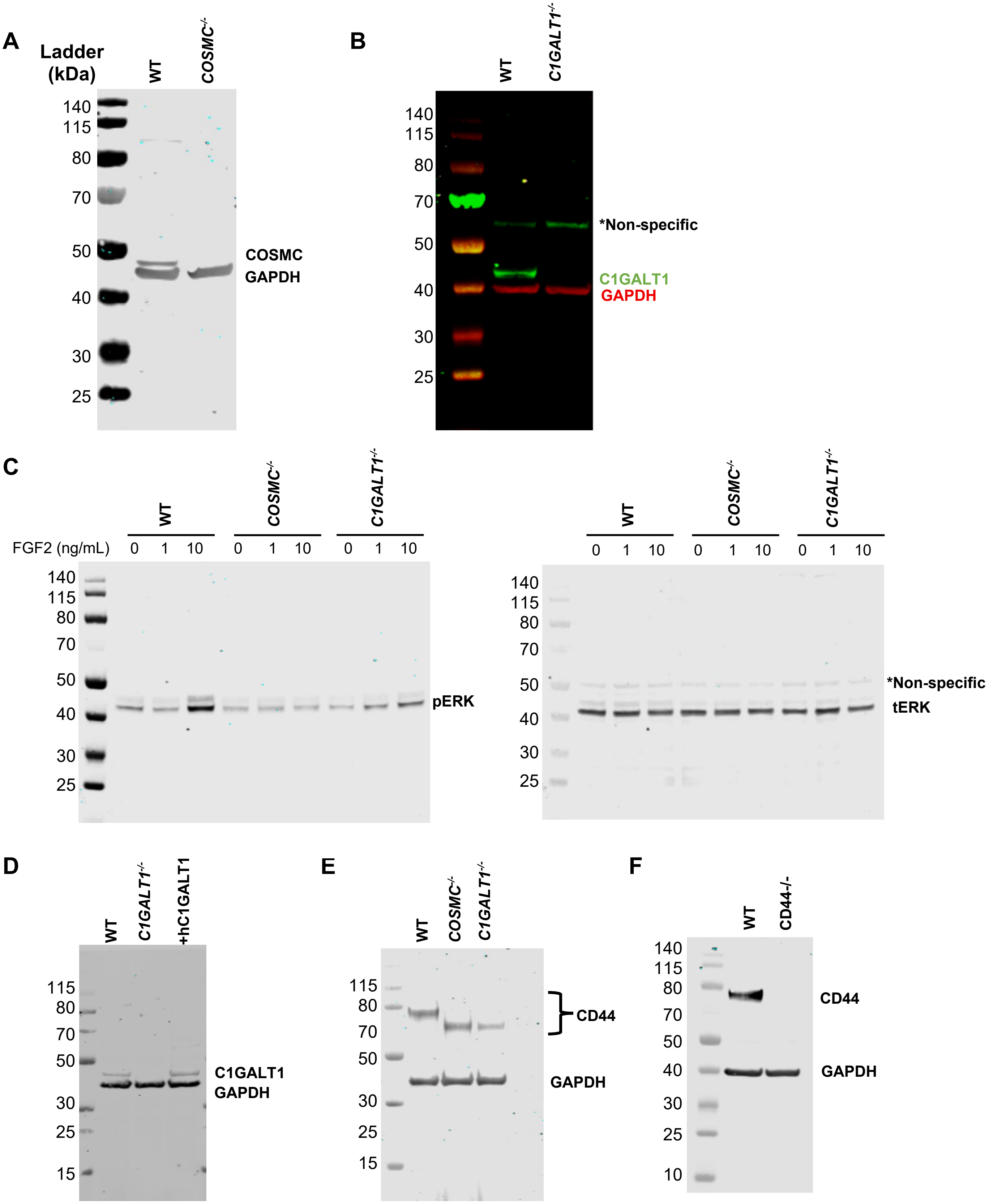

Supplementary Figure 9. Source Data. Uncropped western blot source images for (A) Figure 1B, (B) Figure 1C, (C) Figure 2E, (D) Figure 2G, (E) Figure 6B, and (F) Figure 6F.
