# Supplementary material for: Mucin-type O-glycans regulate proteoglycan stability and chondrocyte maturation
Source: bioRxiv. 2025 Dec 14:2025.12.11.693745. Preprint. [Version 2] doi: 10.64898/2025.12.11.693745 (PMC12710741; doi:10.64898/2025.12.11.693745)
Supplement: 1 [file NIHPP2025.12.11.693745V2-supplement-1.pdf]

bioRxiv preprint doi: https://doi.org/10.64898/2025.12.11.693745; this version posted December 14, 2025. The copyright holder for this preprint (which was not certified by peer review) is the author/funder, who has granted bioRxiv a license to display the preprint in perpetuity. It is made available under aCC-BY 4.0 International license.

Supplementary Figure 1. Validation and characterization of TC28a2 *COSMC*$^{-/-}$ and *C1GALT1*$^{-/-}$ knockout clones. CRISPR sgRNA targeting of human (A) *COSMC* and (B) *C1GALT1* in TC28a2 chondrocytes. Frameshift biallelic mutations in clonal cell lines for Exon 1 of *COSMC* and Exon 2 of *C1GALT1* were confirmed by Sanger sequencing. (C) Flow cytometry analysis of anti-Tn antibody binding to wild-type and *COSMC/C1GALT1* knockout cells (n = 3 independent experiments). (D) Workflow for isolation, purification, enzymatic digestion, aniline

bioRxiv preprint doi: https://doi.org/10.64898/2025.12.11.693745; this version posted December 14, 2025. The copyright holder for this preprint (which was not certified by peer review) is the author/funder, who has granted bioRxiv a license to display the preprint in perpetuity. It is made available under aCC-BY 4.0 International license.

tagging, and LC-MS analysis of glycosaminoglycans. GRIL-LC-MS analysis of (E) HS disaccharides, (F) HS sulfates per disaccharide, and (G) HS sulfation in wild-type, *COSMC*$^{-/-}$, and *C1GALT1*$^{-/-}$ knockout lines (n = 4 independent experiments). GRIL-LC-MS analysis of (H) CS/DS disaccharides and (I) CS/DS sulfation of wild-type and knockout cells (n ≥ 3 independent experiments). Data points are shown as mean ± SD; p-values were determined by student t-test with **** p < 0.0001, *** p < 0.001, ** p < 0.01, * p < 0.05.

Supplementary Figure 2. Validation and characterization of additional TC28a2 *COSMC*^{c6} and *C1GALT1*^{t2} knockout clones. Validation of CRISPR targeting for additional (A) *COSMC* (COSMC^{c6}) and (B) *C1GALT1* (C1GALT1^{t2}) knockout clones. Frameshift biallelic mutations were confirmed by Sanger sequencing. Flow cytometry analysis of (C) PNA lectin, (D) VVA lectin, (E) anti-Tn antibody, (F) 3G10, (G) FGF1, and (H) FGF2 binding in COSMC^{c6}and C1GALT1^{t2} knockout clones compared with wild-type control cells. Data points are shown as mean ± SD (n ≥ 3independent experiments); p-values were determined by one-way ANOVA with Tukey post-test with ****p < 0.0001, *** p < 0.001, ** p < 0.01, * p < 0.05.

Supplementary Figure 3. RNA-seq data analysis and qPCR validation experiments. (A) PCA analysis reveals distinct clustering of TC28a2 wild-type, *COSMC*$^{-/-}$, and *C1GALT1*$^{-/-}$ triplicate samples. (B) Hierarchical clustering and differential expression analysis of triplicate RNA-seq datasets. (C) qPCR validation of gene expression changes in additional COSMC^{c6} and C1GALT1^{t2} knockout clones. Data points are shown as mean ± SD (n = 3 independent experiments); p-values were determined by one-way ANOVA with Tukey post-test with **** p < 0.0001, *** p < 0.001, ** p < 0.01, * p < 0.05.

Supplementary Figure 4. One-step Selective exoenzymatic labeling (SEEL) with ST6GALNAC1. (A) Commasie stained SDS-PAGE gel for purified recombinant human ST6GALNAC1-GFP fusion protein. (B) Schematic of one-step SEEL labeling of TC28a2 cells with

bioRxiv preprint doi: https://doi.org/10.64898/2025.12.11.693745; this version posted December 14, 2025. The copyright holder for this preprint (which was not certified by peer review) is the author/funder, who has granted bioRxiv a license to display the preprint in perpetuity. It is made available under aCC-BY 4.0 International license.

ST6GALNAC1 and CMP-Neu5Ac-C5-triazole-biotin. (C) Representative western blot showing streptavidin enrichment versus flow through from SEEL labeling of *COSMC*$^{-/-}$ and *C1GALT1*$^{-/-}$ cells. The one-step SEEL reactions with ST6GALNAC1 were performed as described in the Methods section. (D) The SEEL labeling method detected a number of known *O*-GalNAcylated glycoproteins (black), including multiple heparan sulfate proteoglycans (blue).

Supplementary Figure 5. Immunofluorescence images of SDC1 staining in TC28a2 chondrocytes. (A) Representative confocal images of SDC1 (red) and nuclei (DAPI; blue) in DMSO-treated cells. The scale bar represents 20 nm. (B) Quantification of SDC1 fluorescence intensity using ImageJ. (C) Representative confocal images of SDC1 (red) and nuclei (DAPI; blue) in cells after treatment with bafilomycin A1 (250 nM) for 16 hours. The scale bar represents 20 nm. (B) Quantification of SDC1 fluorescence intensity in bafilomycin A1-treated cells versus wild-type controls. Data points are shown as mean ± SD (n = 25-35 cells from three independent biological replicates); p-values were determined by one-way ANOVA with Tukey post-test with **** $p < 0.0001$.

Supplementary Figure 6. Anti-CD44 binding assays and *CD44*$^{-/-}$ knockout clone validation. (A) Flow cytometry analysis of cell surface CD44 levels in *COSMC*^{c6} and *C1GALT1*^{t2} knockout clones. (B) Time course quantification of cell surface CD44 levels via flow cytometry prior to and after trypsinization (0-24 hours). Data is normalized to untreated wild-type cells. Data points are shown as mean ± SD (n = 3 independent experiments); p-values were determined by one-way ANOVA with Tukey post-test with **** $p < 0.0001$, *** $p < 0.001$, ** $p < 0.01$, * $p < 0.05$. (C) CRISPR sgRNA targeting of human *CD44* in TC28a2 chondrocytes. (C) Frameshift biallelic mutations in a clonal cell line for Exon 5 of *CD44* were confirmed by Sanger sequencing.

Supplementary Figure 7. Validation of murine GPLC *Cosmc*$^{-/-}$ and *C1galt1*$^{-/-}$ knockout clones. CRISPR sgRNA targeting of mouse (A) *Cosmc* and (B) *C1galt1* in murine growth plate-

bioRxiv preprint doi: https://doi.org/10.64898/2025.12.11.693745; this version posted December 14, 2025. The copyright holder for this preprint (which was not certified by peer review) is the author/funder, who has granted bioRxiv a license to display the preprint in perpetuity. It is made available under aCC-BY 4.0 International license.

like chondroprogenitors (GPLCs). Frameshift biallelic mutations in a clonal cell line for Exon 1 of *Cosmc* and Exon 2 of *C1galt1* were confirmed by Sanger sequencing.

bioRxiv preprint doi: https://doi.org/10.64898/2025.12.11.693745; this version posted December 14, 2025. The copyright holder for this preprint (which was not certified by peer review) is the author/funder, who has granted bioRxiv a license to display the preprint in perpetuity. It is made available under aCC-BY 4.0 International license.

Suppl Fig 1

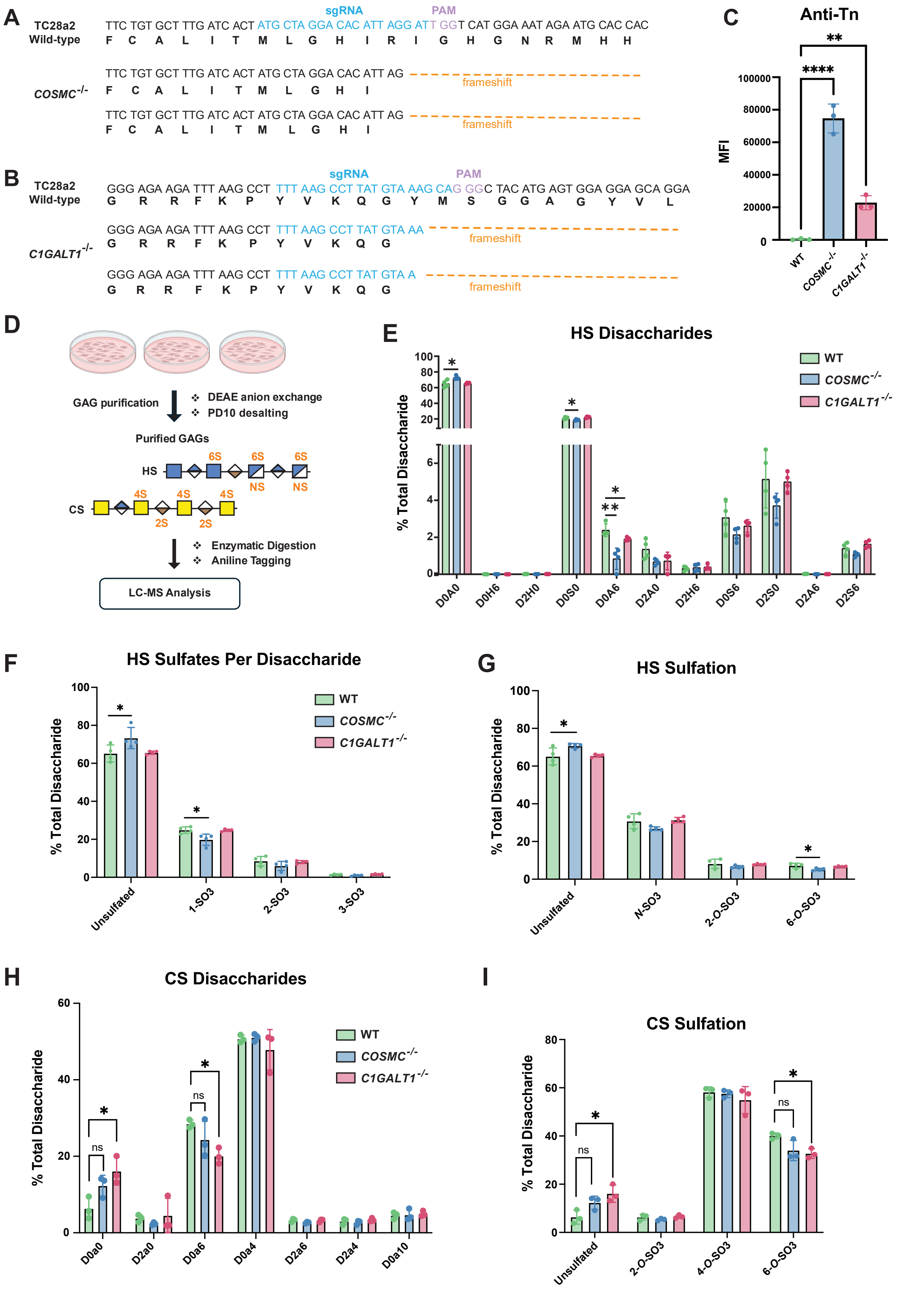

bioRxiv preprint doi: https://doi.org/10.64898/2025.12.11.693745; this version posted December 14, 2025. The copyright holder for this preprint (which was not certified by peer review) is the author/funder, who has granted bioRxiv a license to display the preprint in perpetuity. It is made available under aCC-BY 4.0 International license.

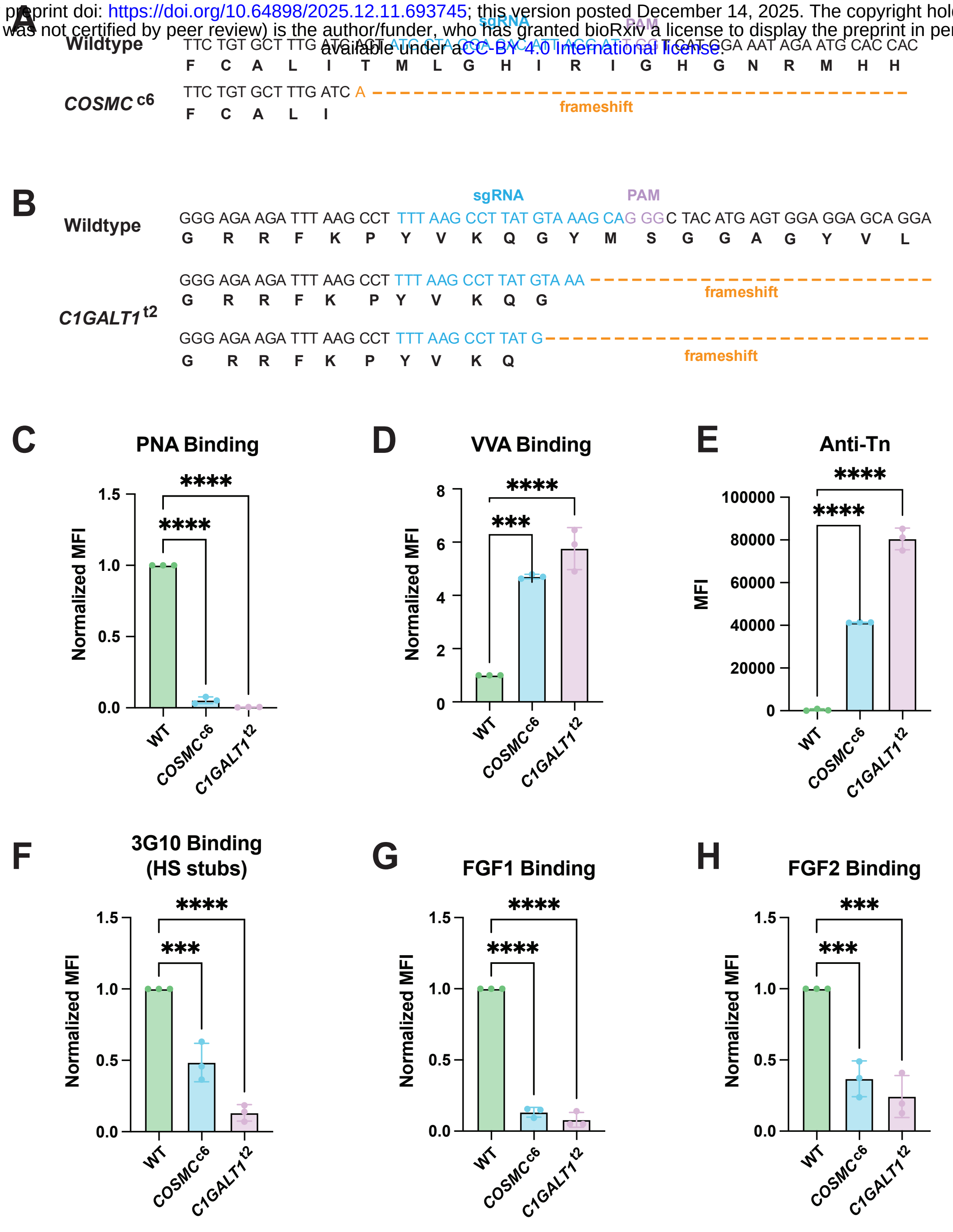

bioRxiv preprint doi: https://doi.org/10.64898/2025.12.11.693745; this version posted December 14, 2025. The copyright holder for this preprint (which was not certified by peer review) is the author/funder, who has granted bioRxiv a license to display the preprint in perpetuity. It is made available under aCC-BY 4.0 International license.

Suppl Fig 3

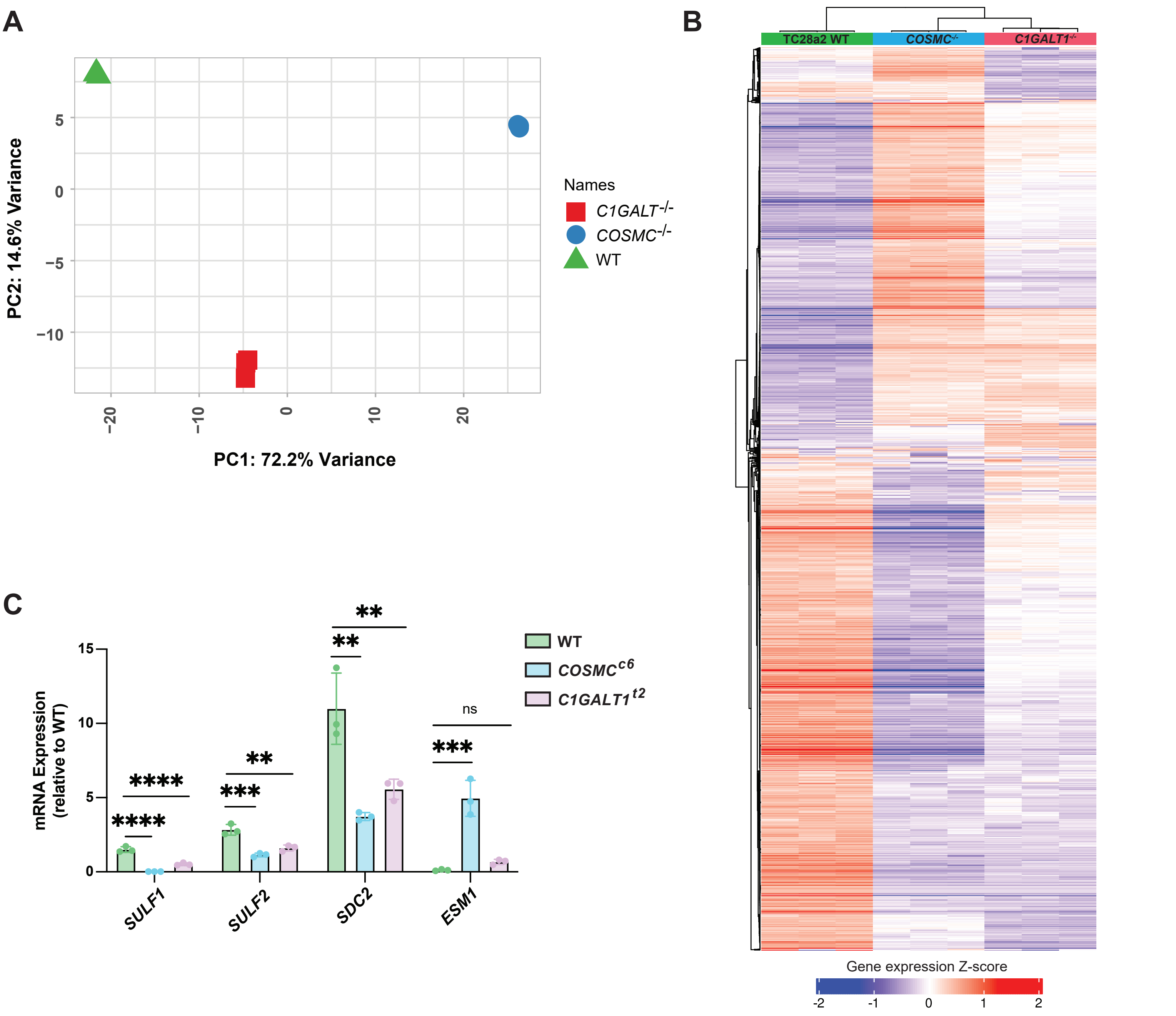

Suppl Fig 4

bioRxiv preprint doi: https://doi.org/10.64898/2025.12.11.693745; this version posted December 14, 2025. The copyright holder for this preprint (which was not certified by peer review) is the author/funder, who has granted bioRxiv a license to display the preprint in perpetuity. It is made available under aCC-BY 4.0 International license.

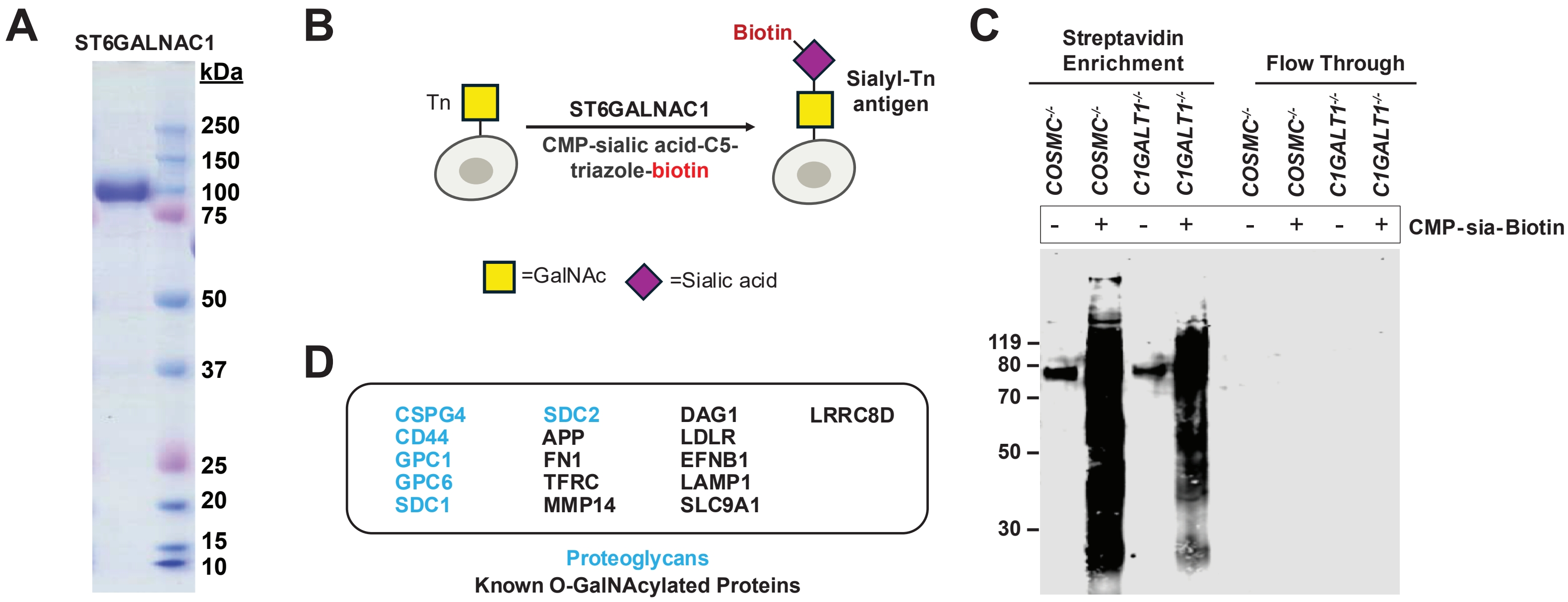

bioRxiv preprint doi: https://doi.org/10.64898/2025.12.11.693745; this version posted December 14, 2025. The copyright holder for this preprint (which was not certified by peer review) is the author/funder, who has granted bioRxiv a license to display the preprint in perpetuity. It is made available under aCC-BY 4.0 International license.

Suppl Fig 5

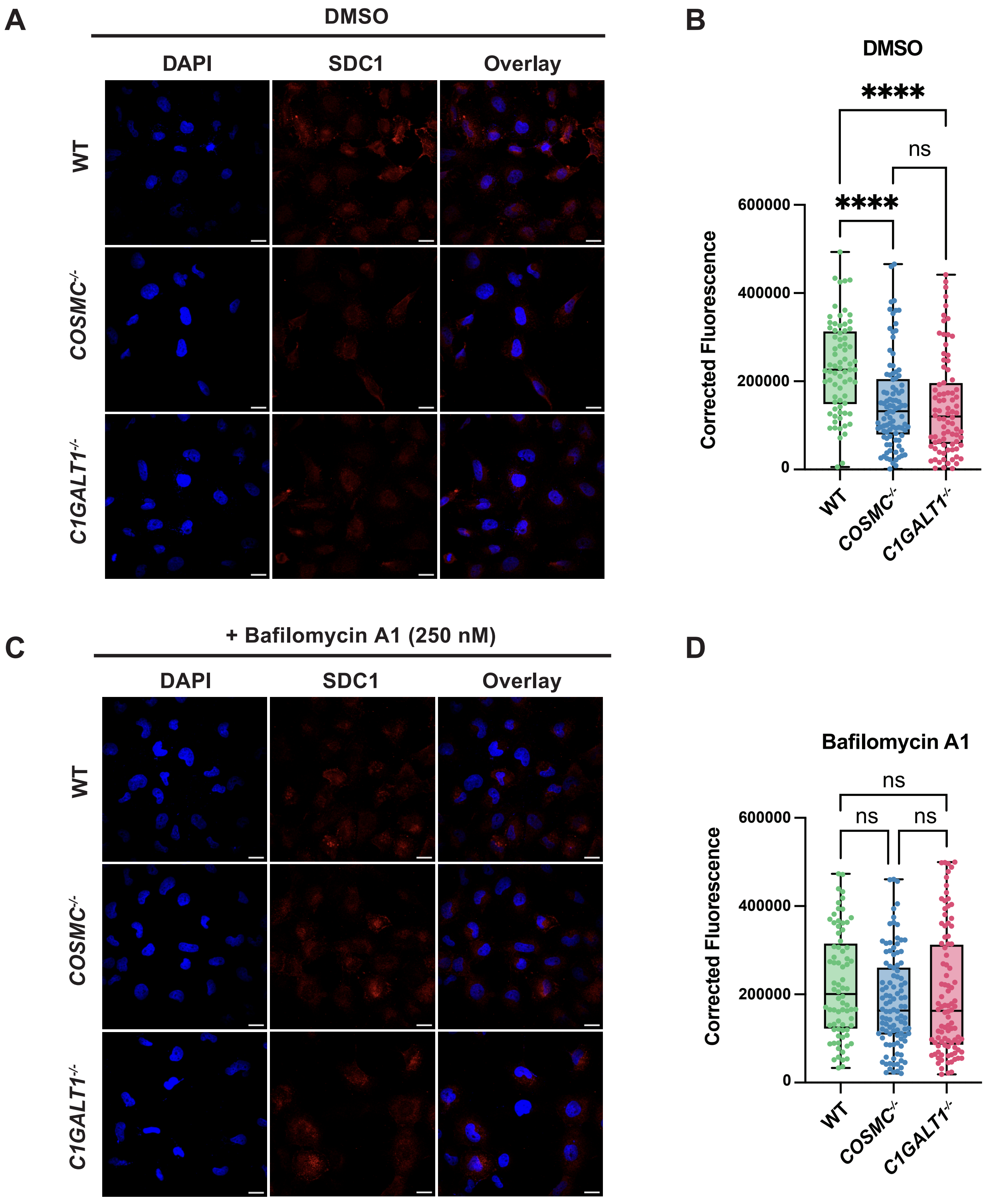

bioRxiv preprint doi: https://doi.org/10.64898/2025.12.11.693745; this version posted December 14, 2025. The copyright holder for this preprint (which was not certified by peer review) is the author/funder, who has granted bioRxiv a license to display the preprint in perpetuity. It is made available under aCC-BY 4.0 International license.

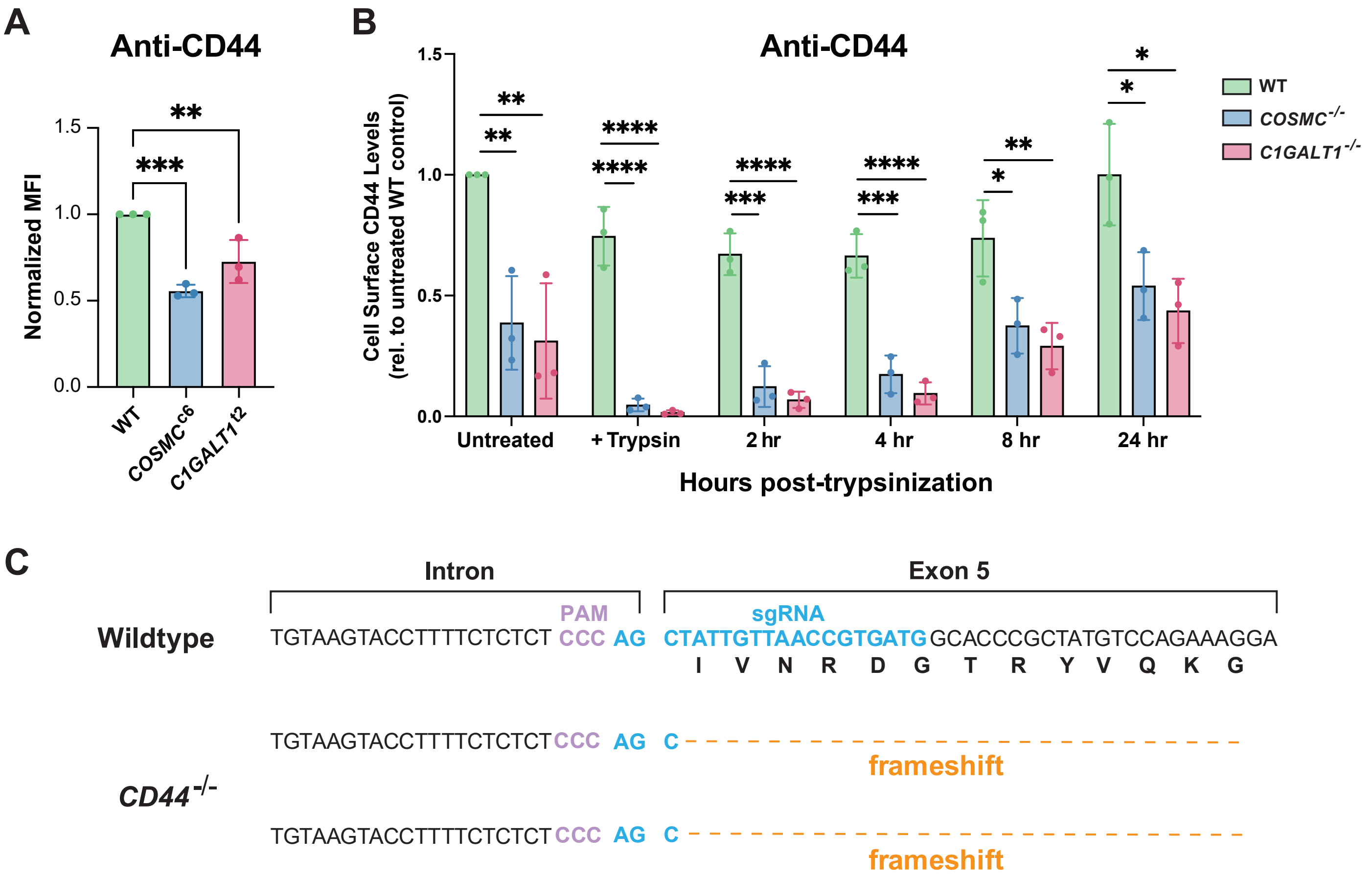

bioRxiv preprint doi: https://doi.org/10.64898/2025.12.11.693745; this version posted December 14, 2025. The copyright holder for this preprint (which was not certified by peer review) is the author/funder, who has granted bioRxiv a license to display the preprint in perpetuity. It is made available under aCC-BY 4.0 International license.

A

sgRNA PAM

GPLC WT

GTG TTT GAG TCA ATT AAT ATG GAC ACA AAT GAC ATG TGG TTG ATG ATG AGG AAA GCT

V F E S I N M D T N D M W L M M R K A

Cosmc$^{-/-}$

GTG TTT GAG TCA ATT A AT ATG GAC ACA AAT G - - - - - - - - - - frameshift

V F E S I N M D T N

B

sgRNA PAM

GPLC WT

CGA AGA TTT AAG CCC TAT GTG AAG CAG GGA TAC ATG AGC GGA GGA GCG GGC TAT GTC

R R F K P Y V K Q G Y M S G G A G Y V

C1galt1$^{-/-}$

CGA AGA TTT AAG CCC TAT GTG AAG CAG GGA TAC AT - - - - - - - - - - frameshift

R R F K P Y V K Q G Y
